# Artepillin C Time−Dependently Alleviates Metabolic Syndrome in Obese Mice by Regulating CREB/CRTC2−BMAL1 Signaling

**DOI:** 10.3390/nu15071644

**Published:** 2023-03-28

**Authors:** Lei Wang, Lingqin Zhou, Shuai Liu, Yaxin Liu, Jia Zhao, Yaqiong Chen, Yi Liu

**Affiliations:** 1 Key Laboratory of Metabolism and Molecular Medicine of the Ministry of Education, Department of Biochemistry and Molecular Biology, School of Basic Medical Sciences, Fudan University, Shanghai 200032, China; 2 Key Laboratory of Medical Neurobiology and MOE Frontier Center for Brain Science, Institutes of Brain Science, Fudan University, Shanghai 200032, China; 3 Key Laboratory of Metabolism and Molecular Medicine of the Ministry of Education, Department of Endocrinology and Metabolism, Zhongshan Hospital, Fudan University, Shanghai 200032, China

**Keywords:** artepillin C, BMAL1, circadian rhythm, liver, glucose and lipid metabolism, metabolic syndrome

## Abstract

Artepillin C (APC), a cAMP-response element−binding (CREB)/CREB regulated transcription coactivator 2 (CRTC2) inhibitor isolated from Brazilian green propolis, can ameliorate metabolic syndrome in obese mice. Because the sensitivity and responsiveness of the body to the drug depend on the time of day and the circadian clock alignment, the optimal administration time of APC for desired efficacy in treating metabolic syndrome remains unclear. In this study, APC (20 mg/kg) or the vehicle was intraperitoneally injected into obese mice once daily for one or three weeks. The results of the insulin tolerance test, pyruvate tolerance test, and histological and biochemical assays showed that APC could improve whole−body glucose homeostasis and decrease hepatic lipid synthesis following a circadian rhythm. Further exploration of the underlying mechanism revealed that APC may disturb the diurnal oscillations of the expression of brain and muscle ARNT−like protein (BMAL1) in primary hepatocytes and the livers of the study subjects. Moreover, APC could inhibit hepatic BMAL1 expression by blocking the CREB/CRTC2 transcription complex. BMAL1 overexpression in primary hepatocytes or the livers of *db/db* mice antagonized the inhibitory effect of APC on hepatic lipid metabolism. In conclusion, the chronotherapy of APC may relieve metabolic syndrome in obese mice, and the mechanism behind APC−mediated time−of−day effects on metabolic syndrome were unveiled, thereby providing a foundation for optimized APC treatment from a mechanistic perspective.

## 1. Introduction

Artepillin C (APC) is a major component of Brazilian green propolis with a wide range of pharmacological benefits, including antioxidant [1,2], anti−inflammatory [3,4], anticancer [5,6], and immunomodulatory effects [6,7]. Our previous report demonstrated that APC could ameliorate metabolic syndrome by suppressing CREB/CRTC2− mediated gluconeogenesis and the transcription of sterol regulatory element−binding protein (SREBP) in obese mice [8]. It is well−established that the drug efficiency and toxicity can oscillate over a 24 h period in which the circadian clock plays a pivotal role [9,10]. Despite the well−defined benefits of APC on glucose and lipid metabolism, a comprehensive understanding of the impact of the administration time on the optimal effects of APC is still elusive.

The circadian clock aligns physiological and behavioral processes to the 24 h self−rotation of the Earth. It consists of a central clock in the hypothalamic suprachiasmatic nuclei (SCN) and several peripheral clocks in peripheral tissues (e.g., liver, fat, muscle, and heart) [11,12,13]. Accumulating evidence has revealed that multiple clock genes participate in up to 80% of gene expression that mediates metabolism and metabolic homeostasis in mammals [14,15,16]. In addition, metabolic diseases may occur if the circadian rhythms are disrupted [17,18,19], suggesting a close correlation between glucose/lipid metabolism and the circadian clock.

The circadian clock’s molecular components in mammals form a cell−autonomous transcriptional translational feedback loop (TTFL) [20,21] in which BMAL1 and CLOCK complex into heterodimers to regulate the expression of their repressors: cryptochrome (Cry1 and Cry2) and period (Per1, Per2 and Per3) genes, respectively [22]. Additionally, nuclear hormone receptors RORs and REV−ERBs can repress or activate BMAL1 expression, thereby forming a second feedback loop [23]. BMAL1 has been reported to closely couple with fundamental metabolic processes [24,25,26]. For example, mice with liver−specific BMAL1 deletion exhibit hypoglycemia, exaggerated glucose clearance problems, and disturbed rhythmic expression of regulatory genes for hepatic glucose [27].

Based on the previous literature, we considered the circadian clock to participate in APC−mediated inhibition of lipid and glucose metabolism in obese mice. In this study, APC was found to improve glucose and lipid homeostasis by suppressing Bmal1 transcriptional activity via blockade of CREB/CRTC2−BMAL1 signaling. In addition, our results demonstrated that APC alleviated metabolic syndrome in obese mice in a circadian rhythmic manner, thereby providing a mechanistic basis for optimizing APC administration.

## 2. Materials and Methods

### 2.1. Animals and Experimental Design

C57BL/6J wild type, DIO and *db/db* mice were obtained from Fudan University Animal Centre (Shanghai, China), housed in a cage with a light/dark cycle of 12 h/12 h, and provided with water and food ad libitum during the housing period. For high−fat feeding studies, a 60% fat diet (Research Diets, New Brunswick, NJ, USA, D12492) was given to 6−week−old male C57BL/6J mice for 13 weeks. This study was approved by the institutional research ethics committee (No. 202011023S), and all procedures complied with the Guide of the Animal Care and Use Committee of Fudan University.

The obese mice were randomly assigned to the APC group or the control group. The APC group was subsequently intraperitoneally administered with 20 mg/kg APC in 5% DMSO, 40% PEG 300 (Selleck, Washington, DC, USA, S6704), 5% Tween 80 (Selleck, USA, S6702), and 50% PBS buffer daily for 1 or 2 weeks on Zeitgeber time 5 (ZT5) or ZT17, and the control group was administered with the equivalent vehicle. Three hours after the treatment, all the mice were analyzed at ZT8 or ZT20. Both groups were sacrificed at given times (ZT8 or ZT20) after the last dose, and the samples were collected. At ZT20, all the experiments of mice were performed under the dark condition, including APC or vehicle injection, blood glucose testing, and sample collection.

For BMAL1 rescuing studies, 1 × 10^8^ pfu of Ad−GFP or Ad−BMAL1 adenovirus was employed. The 8−week−old male C56BL/6J mice were intraperitoneally administered with vehicle or APC (20 mg/kg) for 3 days. After that, 1 × 10^8^ pfu of Ad−GFP or Ad−BMAL1 adenovirus were dissolved in 100 μL saline and delivered to the above mice by tail vein injection. Then, mice were subjected to experiments after 2 weeks viral expression and APC or vehicle injection. APC was synthesized following a published method [8].

### 2.2. Cell Culture

HEK293T cells obtained from ATCC and DMEM (Gibco, Grand Island, NY, USA, 8117254) were supplemented with 10% fetal bovine serum (FBS) (Gibco, US) and 1% penicillin/streptomycin (Gibco, US, 15140122) which were used to culture the cells. Mouse primary hepatocytes were prepared according to a previous report [28] and cultured in Medium 199 (Hyclone, Logan, UT, USA, AC10210633) with the same supplements as the DMEM. Lipofectamine 2000 (ThermoFisher, Waltham, MA, USA, 11668019) was used to transfect the HEK293T cells following the manufacturer’s recommended protocols [29]. Adenovirus infection to the primary hepatocytes was performed for 24 h before experiments.

### 2.3. Luciferase Activity Assays

For cell luciferase activity assays, HEK293T cells were cultured in several 24−well plates before being transfected with Bmal1−luciferase reporter plasmids (400 ng) and RSV−β−gal plasmids (100 ng) using PEI and DMSO or APC (30 μM) overnight and being induced by forskolin (10 nM) for 7 h. Mouse primary hepatocytes were infected with adenoviruses Bmal1−luciferase reporter together with DMSO or APC (30 μM) for 24 h and induced by glucagon (100 nM) for 7 h. Following the induction, cells were collected and detected following a published method [8].

For liver luciferase activity assays, 5 × 10^7^ pfu Ad−RSV−β−gal and 1 × 10^8^ pfu Ad−Bmal1−lucadenoviruses were injected into mice from the tail vein after the intraperitoneal injection of vehicle or APC (20 mg/kg) on a daily basis for 1 week. After 3–5 days of adenovirus expression, sterile firefly D−luciferin (Biosynth AG, Swiss) at a concentration of 100 mg/kg was injected intraperitoneally into the animals. Then, 3 to 5 min later, an imaging system (IVIS−100) was used to observe the mice. Liver lysates were collected to measure the β−gal activity, which was in turn used to normalize the liver luciferase activity in each well [8].

### 2.4. Isolation of Total RNA Isolation and Quantitative RT−PCR Analysis

Total RNAs from mice tissues and cultured cells were isolated using TRIzol Reagent (Life Technologies, USA, 210807) and purified by the RNA Clean Kit (TIANGEN, China, DP412). The PrimeScript RT reagent kit with a gDNA Eraser (Takara, Japan, RR047A) was utilized to synthesize cDNA. The StepONEPlus Real−Time PCR system (Applied Biosystems, USA) and TB Green^®^ Premix Ex Taq™ (Takara, Japan, RR420A) were utilized to perform the real−time PCR following the manufacturer’s protocols. The primers are given in Table S1.

### 2.5. Protein Extraction and Western Blotting

Mice tissues and cultured cells were lysed in RIPA buffer with proteinase inhibitors (PMSF, Cocktail and Phosphatase Inhibitors). Protein concentrations were determined by using the BCA assay kit (Pierce, USA, 23225) according to the manufacturer’s protocols. After the BCA assay, proteins went through SDS−PAGE electrophoresis and were transferred to PVDF membranes (Millipore, USA) activated with methanol. Subsequently, the proteins were incubated with primary and secondary antibodies for 16 h at 4 °C and for 1 h at ambient temperature. Finally, ECL Western blotting substrate (Share−Bio, China) was implemented to detect the protein bands. Table S2 gives the primary antibodies used in these experiments.

### 2.6. Plasmid

The HA−BMAL1, FLAG−CRTC2,and MYC−CREB plasmids were generated using previously published procedures [30]. The *Bmal1*−Luc plasmids were also used as described in the literature [31].

### 2.7. Adenoviruses

Adenoviruses expressing BMAL1, CRTC2, GFP, *Bmal1*−Luc, and RSV−β−gal were generated by homologous recombination of pAD−Track, a linearized transfer vector, and pAD−Easy, an adenoviral backbone vector [32]. The Ad−BMAL1 adenoviruses were used as described in the literature [28].

### 2.8. Immunofluorescence Staining

Mice livers were collected and fixed in 4% PFA for 24–48 h before being embedded in OCT compound (Sakura, Japan) and sliced into frozen sections with sizes of 8–10 μm. The sections were blocked in PBS with 0.1% Triton X−100 and 5% bovine albumin (BSA) for 30 min at ambient temperature. Subsequently, the samples were incubated with primary antibodies in 5% BSA overnight and with secondary antibodies (diluted 1:1000 in 5% BSA) for 1 h at room temperature. Finally, the slides were counterstained with Hoechst for 15 min and mounted in 80% glycerol before imaging.

### 2.9. Haematoxylin and Eosin Staining

Liver tissues were fixed in 4% PFA for 24–48 h and embedded in OCT (Sakura, Japan), and hematoxylin and eosin (Solarbio, China, G1120) were used to stain the slices.

### 2.10. Pyruvate Tolerance Tests (PTT) and Insulin Tolerance Tests (ITT)

For PTT, the mice were fasting for 16 h before ip. injected with sodium pyruvate (1–2 g/kg). About ITT, insulin (1–2 U/kg) was injected intraperitoneally into mice after 3 h fasting. The glucose concentration in the blood was measured at 0, 15, 30, 60, and 120 min after pyruvate or insulin administration.

### 2.11. Plasma Lipid Assay

The total cholesterol, LDL cholesterol, TG, and plasma glycerol levels were measured with commercial kits (Jiancheng, China).

### 2.12. Lipid Production Assay

Ad−GFP or Ad−BMAL1 were used to infect the primary hepatocytes for 24 h, and these cells were exposed to APC for 1 h prior to 18 h insulin (100 nM) stimulation. The total cholesterol and TG levels in primary hepatocytes were detected with commercial kits (Jiancheng, China).

### 2.13. Statistical Analyses

Image J and GraphPad Prism 8 were utilized to perform statistical analyses. All results are presented as the mean ± SEM. Student’s *t*−tests or one−way ANOVA were used to determine the presence of statistical differences. Cosinor was used to analyze the oscillations of BMAL1, FASN, and PEPCK in DMSO− or APC−treated primary hepatocytes; * *p* < 0.05; ** *p* < 0.01; *** *p* < 0.001 for all the figures in the following sections.

## 3. Results

### 3.1. APC Could Enhance Glucose Homeostasis in a Circadian Rhythmic Manner

**Figure 1:**
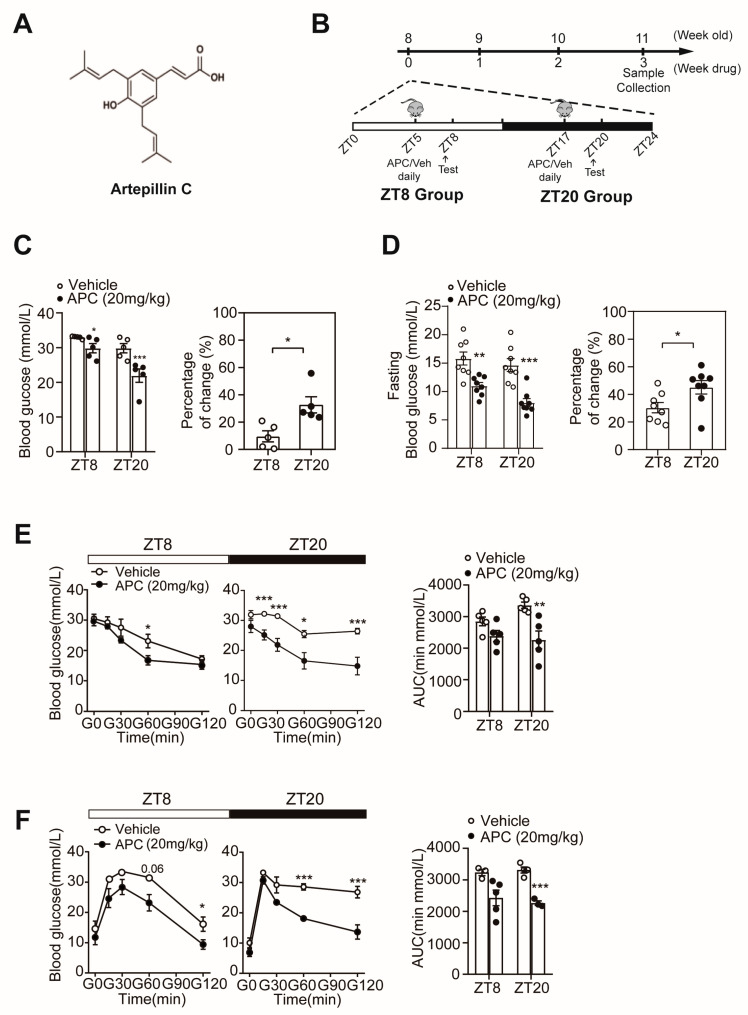
Circadian rhythmic impacts of APC on glucose metabolism in *db/db* mice: (**A**) molecular architectures of artepillin C; (**B**) schematic diagram of experimental scheme; (**C**) ad libitum blood glucose levels at ZT8 or ZT20 in *db/db* mice treated with 20 mg/kg APC or vehicle for 1 week (*n* = 5 per group). The right panel shows the percentage change in blood glucose levels at 2 different times ZT8 and ZT20; (**D**) after fasting 16 h, the blood glucose levels at ZT8 or ZT20 in *db/db* mice treated with 20 mg/kg APC or vehicle for 1 week (*n* = 8 per group). The right panel shows the percentage change in fasting blood glucose levels; (**E**) insulin tolerance test (ITT) and (**F**) pyruvate tolerance test (PTT) were performed around ZT8 and ZT20. The *db/db* mice were administrated APC (20 mg/kg) or vehicle for 2 weeks. The results of area under the curve (AUC) are shown at the bottom of each test curve (*n* = 3–5 per group). * *p* < 0.05; ** *p* < 0.01; *** *p* < 0.001.

Our previous study showed that APC reduces fasting blood glucose in obese mice by interrupting the process to form the CREB/CRTC2 transcriptional complex (Figure 1A) [8]. To further explore whether the administration time of APC alters its effect on glucose homeostasis, mice were administered with APC or vehicle daily at ZT5 or ZT17 and tested at ZT8 or ZT20, respectively (Figure 1B). After 1 week of treatment, the APC treatment group showed reduced glucose levels at ZT8 and ZT20 compared to the vehicle group, and the blood glucose levels at ZT20 were lower than that those at ZT8 (Figure 1C). Moreover, APC was found to reduce fasting glucose levels and enhance insulin sensitivity more significantly at ZT20 (Figure 1D,E). Subsequently, we performed a PTT in *db/db* mice. As shown in Figure 1F, APC treatment was revealed to significantly decrease the AUC of PTT assays compared to the treatment with vehicle, suggesting a significantly lower capacity for hepatic gluconeogenesis. However, such a finding was only present at ZT20 (Figure 1F). Taken together, our results demonstrated the circadian rhythmic impacts of APC on glucose metabolism, providing evidence of the involvement of the circadian clock in physiological processes, especially glucose metabolism and chrono−pharmacology [10,15,33].

### 3.2. APC Decreased Hepatic BMAL1 Expression by Blocking CREB/CRTC2 Complex

**Figure 2:**
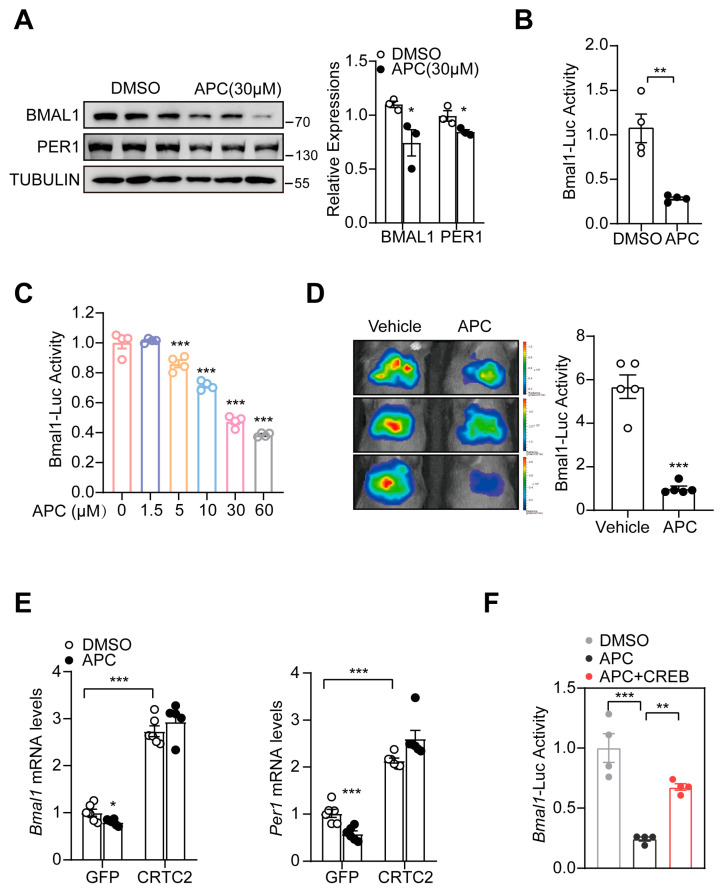
APC inhibits hepatic BMAL1 expression: (**A**) immunoblotting of BMAL1 and PER1 proteins in primary hepatocytes treated with 1 h of DMSO or APC (30 μM) treatment prior to 7 h glucagon stimulation (100 nM, *n* = 3 per treatment); (**B**) luciferase Bmal1−Luc activity; HEK293T cells were infected with Bmal1−Luc and RSV−β−Gal for 24 h, followed by 1 h incubation with APC (30 μM) prior to 7 h forskolin treatment before the luciferase assay (*n* = 4 per treatment); (**C**) primary hepatocytes were infected by AD−Bmal1−Luc and AD−RSV−β−Gal for 24 h, followed by 1 h incubation with APC (0, 1.5, 5, 10, 30, 60 μM) prior to 7 h glucagon stimulation (100 nM, *n* = 4 per treatment) Different colors represent different group treated with different concentrations of APC.; (**D**) live imaging assay of Bmal1−Luc activity in WT mice; the mice were tested at ZT1; (**E**) mRNA levels of Bmal1 and Per1 analyzed by real−time qPCR in primary mouse hepatocytes. The cells were injected with GFP− and CRTC2− expressing adenovirus for 24 h and treated with APC for 1 h prior to 8 h glucagon stimulation (100 nM, *n* = 6 per group); (**F**) luciferase Bmal1−Luc activity; HEK293T cells were infected with Bmal1−Luc, CREB, and RSV−β−Gal for 24 h, then incubated with APC (30 μM) for 1 h prior to forskolin 7 h before luciferase assay (*n* = 4 per treatment). * *p* < 0.05; ** *p* < 0.01; *** *p* < 0.001.

To reveal the molecular foundation of APC’s effects in *db/db* mice, we hypothesized that the circadian clock participates in the APC−mediated glucose metabolism. As the master regulator of the molecular clock, BMAL1 binds to CLOCK and forms a heterodimer, stimulating the expression of repressors (Pers and Crys) to induce their respective activities. In this study, we overexpressed BMAL1, CREB, and CRTC2 in HEK 293T cells, followed by treatment with APC or DMSO to investigate the impacts of APC on BMAL1 expression. In line with our previous results, APC posed no effects on the expression of CREB and CRTC2 but reduced BMAL1 protein expression (Figure S1A). Moreover, APC was identified to cut down the endogenous expression of BMAL1 protein in primary hepatocytes (Figure 2A). Bmal1−Luc, a luciferase reporter driven by the *Bmal1* promoter, was cloned, whose activity was dramatically inhibited following APC treatment in the two cell lines investigated (Figure 2B,C). Higher APC concentrations corresponded to lower Bmal1−Luc activity in primary hepatocytes, indicating that APC may influence Bmal1−Luc expression in a dose−dependent manner (Figure 2C). Moreover, Per1−Luc activity in primary hepatocytes also fell following APC treatment, which conforms to our previous results (Figure S1B,C). As APC was proved to inhibit the expression of BMAL1 in vitro, we moved on to confirm this underlying molecular mechanism in vivo. From liver imaging results, APC was verified to inhibit Bmal1−Luc activity in the livers of mice fasting for 16 h (Figure 2D). CREB, a major transcription factor in eukaryotes, has been revealed to participate in gluconeogenesis and lipid metabolism [30,34,35,36]. As APC may bind to CREB and block the formation of the CREB/CRTC2 complex, we investigated whether APC reduces BMAL1 transcription via this mechanism. CRTC2 proteins were overexpressed in APC−treated primary hepatocytes, and BMAL1 expression was subsequently examined. Compared with the GFP group, CRTC2 overexpression enhanced the expression of BMAL1 mRNA (Figure 2E). The Bmal1−Luc activity was dramatically decreased in the APC group but rescued after CREB overexpression (Figure 2F), which was in line with the notion in Figure 2E.

### 3.3. APC Alters the Circadian Oscillation of BMAL1 Expression in Primary Hepatocytes

**Figure 3:**
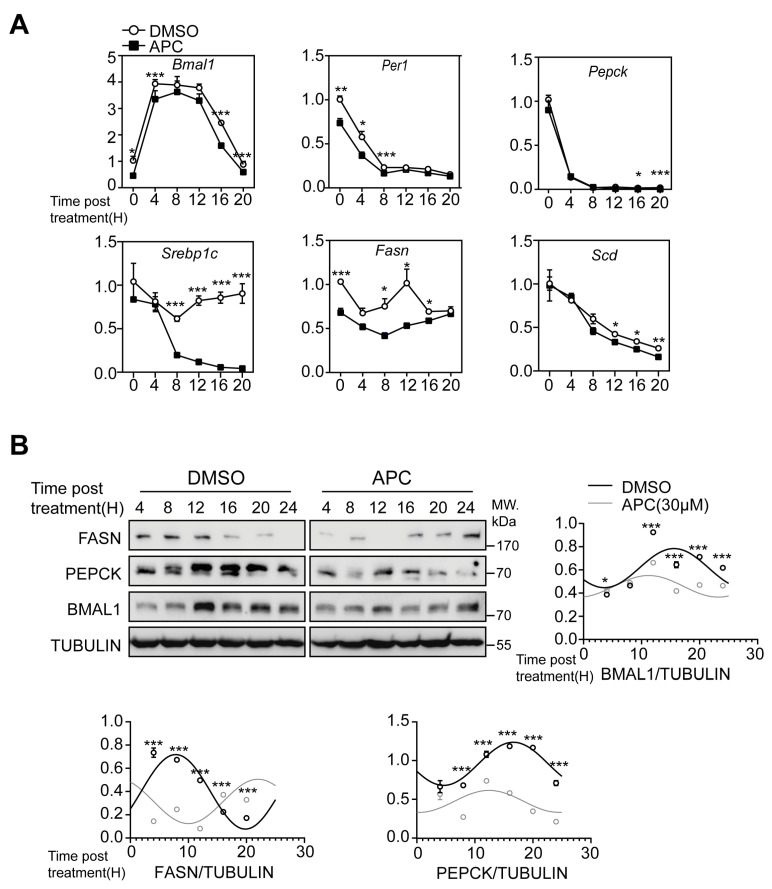
Temporal analysis of hepatic genes expression following APC administration: (**A**) the mRNA levels of the indicated genes analyzed in primary hepatocytes; mouse primary hepatocytes were synchronized by dexamethasone (1 μmol/L) for 2 h and then treated with APC for 1 h prior to 4 h stimulation with glucagon (100 nM, *n* = 3 per group); (**B**) representative Western blots of FASN, PEPCK, BMAL1, and TUBULIN proteins in cells as treated in (**A**). For each sample, the lysates from three replicate wells of primary hepatocytes were pooled together for immunoblot assays. We calculated the means of corresponding densitometry of BMAL1, PEPCK, and FASN protein, and they were superimposed with corresponding sine fittings generated by cosinor analyses. The black circles represent the means of corresponding densitometry of protein in the DMSO group and the grey circles represent the APC group. * *p* < 0.05; ** *p* < 0.01; *** *p* < 0.001.

As circadian clock gene expression has temporal variations, we examined the transcriptional responses of *Bmal1* and *Per1* in primary hepatocytes during a 24 h period. Hepatocytes underwent 1 h DMSO or APC treatment prior to a 4 h glucagon stimulation. Following that, hepatocytes were collected at 4 h intervals over a 24 h period. Compared with samples treated with DMSO, the temporal patterns of *Bmal1* and *Per1* showed a small but statistically significant change in the APC group (Figure 3A). Next, we explored whether APC could influence genes involved in glucose and lipid metabolism and the temporal expression patterns of *Pepck*, *Srebp1c*, *Scd*, and *Fasn* in primary hepatocytes over 24 h. The results showed that APC treatment may alter the temporal expression patterns of these genes (Figure 3A). For BMAL1, PEPCK, and FASN, APC significantly reduced their protein amounts in primary hepatocytes, which is consistent with the inhibitory effects of APC on their corresponding mRNAs (Figure 3B).

### 3.4. APC Rhythmically Modulated Hepatic Glucose and Lipid Metabolism in db/db Mice

**Figure 4:**
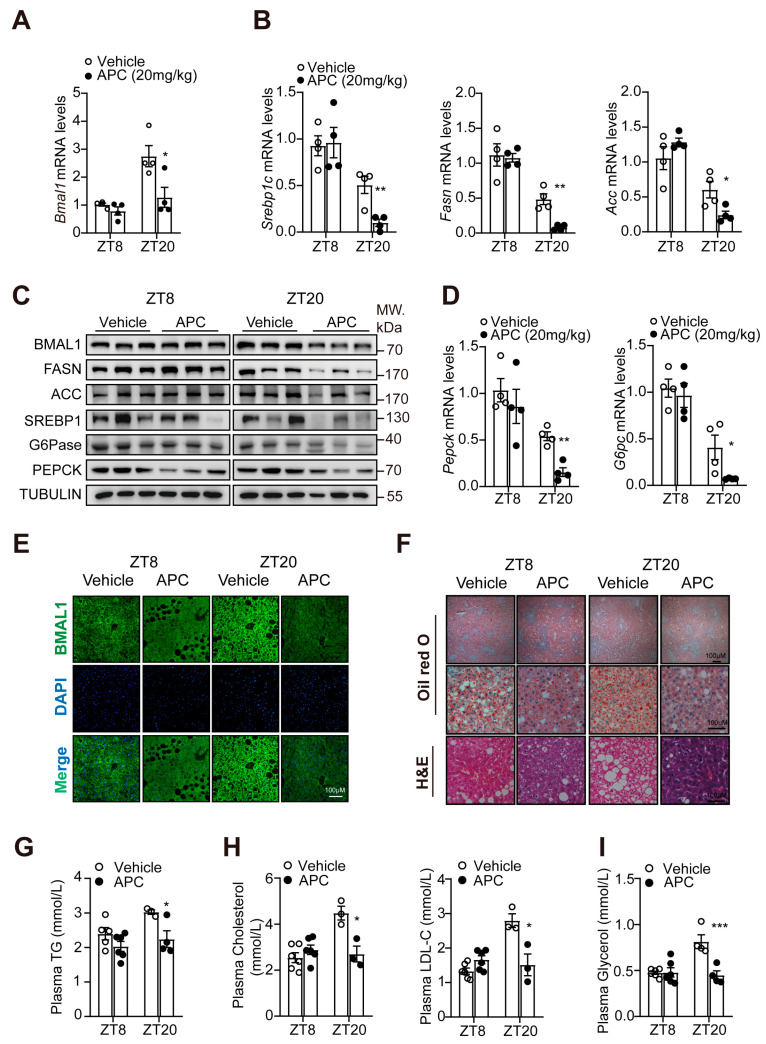
APC rhythmically inhibits hepatic glycolipid metabolism in *db/db* mice: (**A**) mRNA level of *Bmal1* gene analyzed by real−time qPCR in livers; the *db/db* mice were treated by APC (20 mg/kg) or vehicle for 2 weeks (*n* = 4 per group); (**B**,**D**) mRNA levels expression of *Srebp1c*, *Fasn*, *Acc*, *G6pc*, and *Pepck* gene analyzed by real−time qPCR in livers as treated in (**A**) (*n* = 4 per group); (**C**) Western blots of BMAL1, PEPCK, G6pase, ACC, SREBP1, and FASN proteins in livers as treated in (**A**) (*n* = 3 per group); (**E**) immunostaining of BMAL1 in the liver tissues of *db/db* mice; nuclei were stained with DAPI; the scale unit is bar, 100 μM; (**F**) the liver tissues were isolated from *db/db* mice and Oil−red O and H&E staining were performed; the scale unit is bar, 100 μM; (**G**–**I**) plasma TG, TC, LDL−C, and glycerol in the *db/db* mice at two different times at ZT8 and ZT20 (*n* = 3−6 per group). * *p* <0.05; ** *p* < 0.01; *** *p* < 0.001.

In this study, the functional role of APC in regulating the expression of hepatic *Bmal1* and genes relevant to glucose/lipid metabolism was verified in vivo. The *db/db* mice were treated with APC or vehicle for 2 weeks at ZT5 or ZT17 and observed for mRNA and protein expression at ZT8 or ZT20, respectively. Compared to the ZT8 group, the ZT20 group demonstrated that APC could robustly reduce the expression of *Bmal1* mRNA (Figure 4A) and protein (Figure 4C). In addition, APC may decrease the mRNA levels of *Srebp1c*, *Acc*, *Fasn*, *Pepck*, and *G6pc*, as well as the protein levels of BMAL1, FASN, ACC, SREBP1, G6pase, and PEPCK, in the ZT20 group (Figure 4B–D). Immunostaining of BMAL1 in the liver tissues also confirmed that APC may inhibit hepatic BMAL1 in a time−dependent manner (Figure 4E). Correlative evidence suggests that the hepatic lipid metabolism exhibits a diurnal rhythm, the amplitude of which is determined by feeding–fasting cycles [37]. As shown in Figure 4F, the content of hepatic lipid droplets increased in mice treated with vehicle at ZT20 compared to ZT8. However, the increase in hepatic lipid droplet levels observed in the vehicle group was not present in mice treated with APC (Figure 4F). Moreover, APC could significantly reduce the plasma levels of total cholesterol (TC), total triglyceride (TG), glycerol, and low−density lipoprotein cholesterol (LDL−C) at ZT20 (Figure 4G–I). Therefore, these results verified a time−dependent efficacy of APC against metabolic syndrome in *db/db* mice.

### 3.5. Time−Dependent Metabolic Protection of APC in DIO Mice

**Figure 5:**
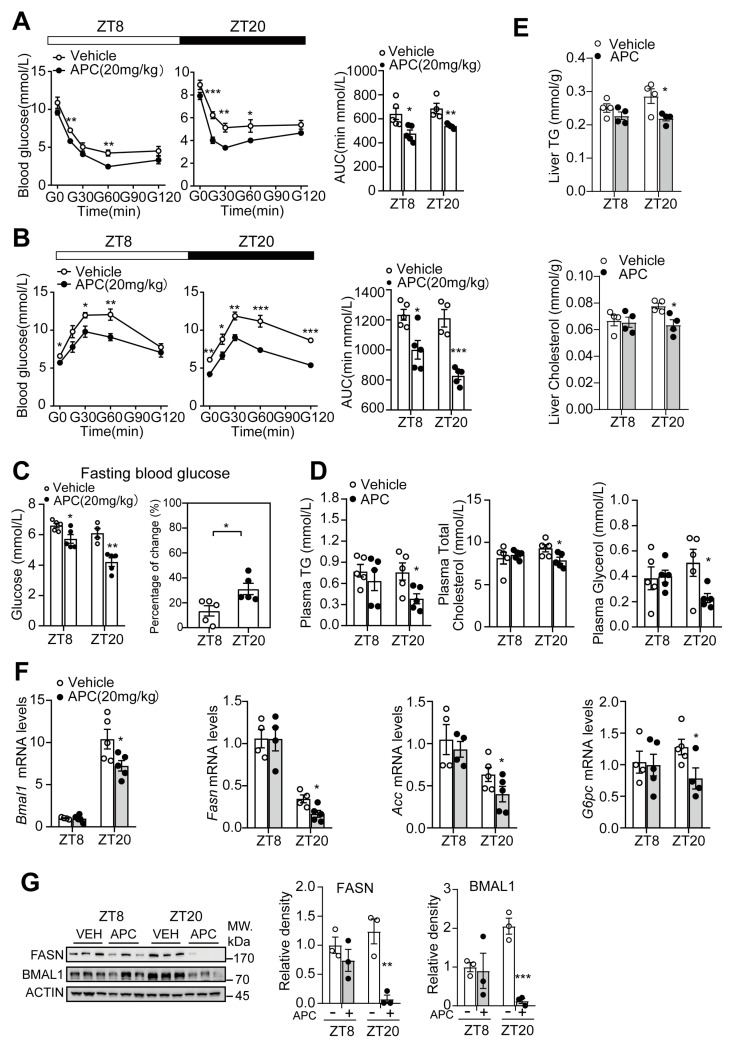
APC improved lipid and glucose homeostasis in DIO mice in a circadian rhythmic manner: (**A**,**B**) ITT and PTT were performed around ZT8 and ZT20. DIO mice were administrated APC or vehicle for 3 weeks. The results of area under the curve (AUC) are shown at the bottom of each test curve (*n* = 5 per group); (**C**) the fasting blood glucose; DIO mice were treated by APC (20 mg/kg) or vehicle for 3 weeks (*n* = 5 per group); after fasting for 16 h, the animals’ blood glucose was tested at ZT8 or ZT20; (**D**) the contents of plasma triglyceride (TG), plasma total cholesterol, and plasma glycerol in the DIO mice (*n* = 5 per group); (**E**) the hepatic TG and TC levels in the DIO mice (*n* = 4 per group); (**F**) mRNA levels expression of *Bmal1*, *Fasn*, *Acc,* and *G6pc* genes analyzed by real−time qPCR in the livers. DIO mice were treated by APC (20 mg/kg) or vehicle for 4 weeks (*n* = 5 per group); (**G**) Western blots of BMAL1 and FASN proteins in livers; the *db/db* mice were treated as (**F**). * *p* <0.05; ** *p* < 0.01; *** *p* < 0.001.

APC treatment could reduce fasting glucose levels (Figure 5C), improve insulin sensitivity (Figure 5A), decrease the capacity of hepatic gluconeogenesis (Figure 5B), reduce plasma TG, TC and glycerol levels, and reduce hepatic TG and TC levels in DIO mice (Figure 5D,E). Similar to the results found in *db/db* mice, the ZT20 group exhibited a marked response to APC, compared to the ZT8 group (Figure 5). Subsequently, we checked the expression of relevant genes in the circadian clock and metabolism. As shown in Figure 5F,G, APC was found to remarkably reduce the mRNA levels of *Bmal1*, *G6pc*, *Acc*, and *Fasn*, as well as the protein levels of BMAL1 and FASN, in the ZT20 group, and these results were consistent with those observed in *db/db* mice. Taken together, these data help verify that APC suppresses BMAL1 expression and glucose and lipid metabolism in a circadian rhythmic manner. From this finding, APC could possibly regulate lipid metabolism by suppressing the transcriptional activity of BMAL1 in the liver.

### 3.6. Involvement of BMAL1 in APC−Mediated Regulation of Hepatic Lipid Synthesis

**Figure 6:**
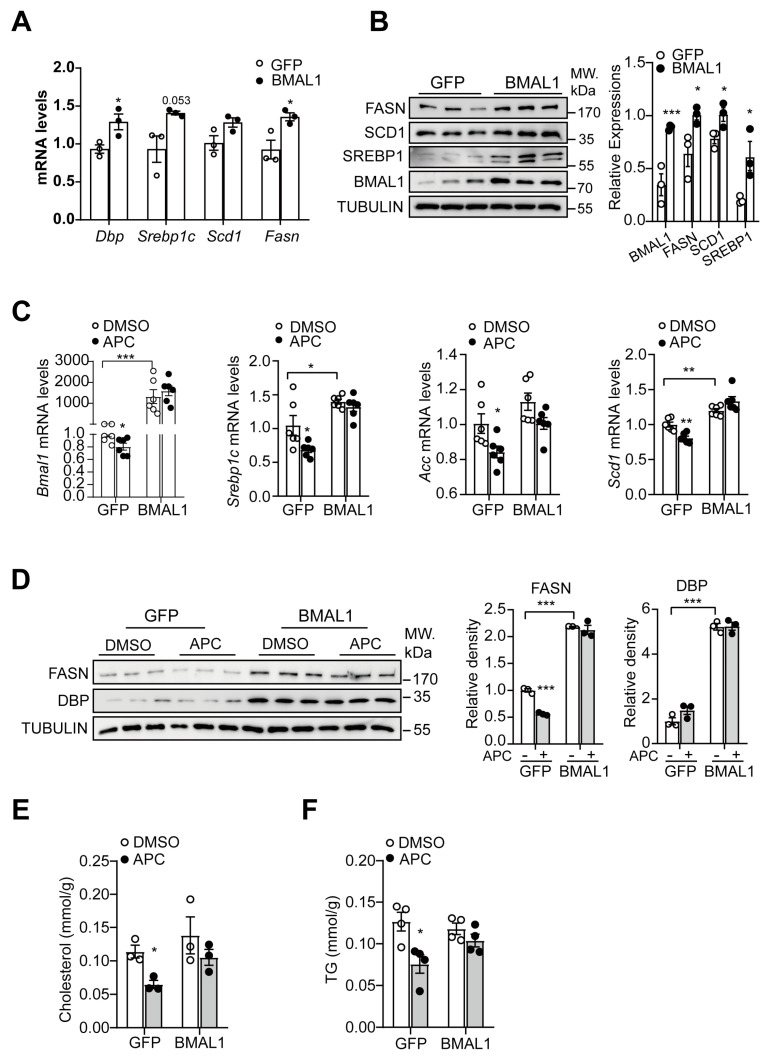
APC inhibits lipid synthesis with an effect dependent on BMAL1: (**A**) mRNA levels of *Dbp* (D site−binding protein), *Srebp1c*, *Scd1*, and *Fasn* analyzed by real−time qPCR in primary hepatocytes. The cells were infected with GFP− and BMAL1− expressing adenovirus for 24 h (*n* = 3 per group); (**B**) Western blots of BMAL1, SREBP1, SCD1, and FASN proteins in primary hepatocytes; the cells were treated as before (*n* = 3 per group); (**C**) mRNA levels of *Bmal1*, *Srebp1c*, *Acc*, and *Scd1* analyzed by real−time qPCR in primary hepatocytes; the cells were infected with GFP− and BMAL1−expressing adenovirus for 24 h and treated with APC for 1 h prior to 18 h insulin stimulation (100 nM, *n* = 6 per group); (**D**) Western blots of DBP and FASN proteins in primary hepatocytes treated the same as (**C**); (**E**,**F**) the contents of TC and TG in primary hepatocytes treated as before (*n* = 3 per group). * *p* < 0.05; ** *p* < 0.01; *** *p* < 0.001.

Our previous report showed that APC improves glucose homeostasis by blocking the CREB/CRTC2 transcriptional complex [8], a process extensively investigated in glucose homeostasis [38]. Previous studies have also suggested a critical role for CREB/CRTC2 in lipid homeostasis [39,40]. Based on these conclusions, we examined whether APC regulates lipid synthesis by suppressing the CREB/CRTC2−BMAL1 signaling pathway. Ectopic BMAL1 expression elevated mRNA and protein levels of those genes associated with lipid synthesis in primary hepatocytes (Figure 6A,B). In primary hepatocytes with BMAL1 overexpression, APC failed to inhibit the transcription of relevant genes in lipid synthesis (Figure 6C,D). Moreover, BMAL1 overexpression counteracted the APC−mediated inhibition of lipid synthesis in primary hepatocytes, suggesting the necessity of BMAL1 for APC to reduce lipid synthesis in primary hepatocytes (Figure 6E,F). 

### 3.7. BMAL1−Overexpression in the Liver Antagonizes APC−Mediated Inhibition in Hepatic Lipid Synthesis

**Figure 7:**
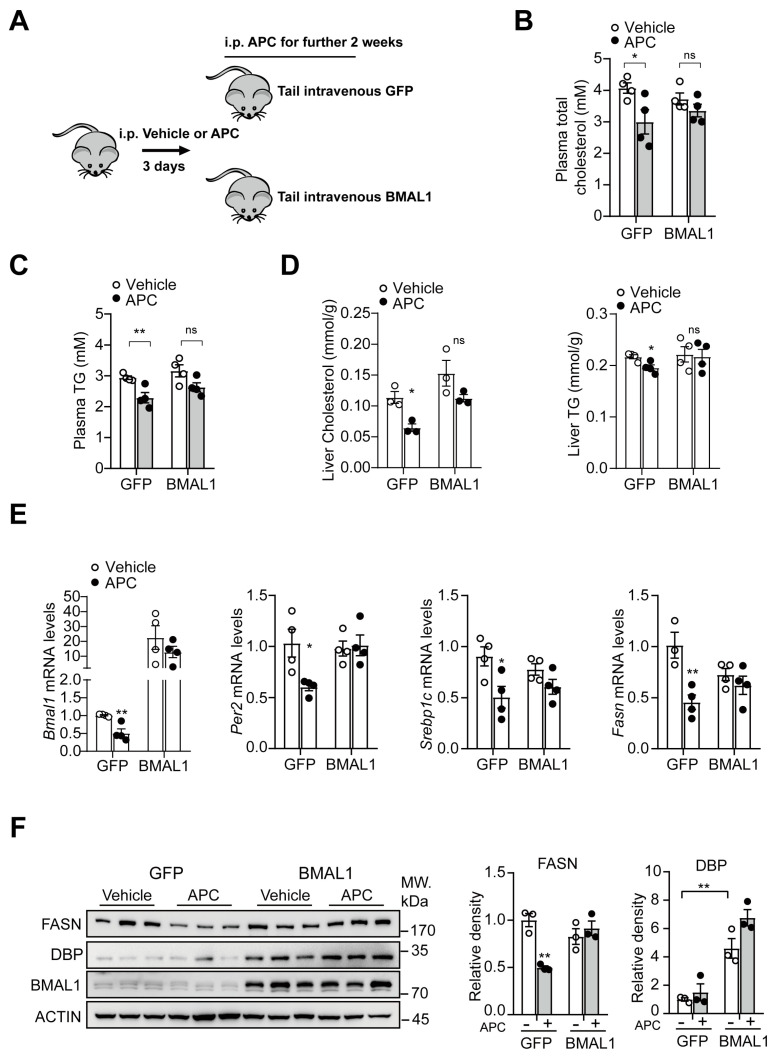
In *db/db* mice, APC inhibits lipid synthesis in a BMAL1−dependent manner at ZT20: (**A**) schematic description of animal experiments. The mice were treated with vehicle or APC (20 mg/kg) at ZT17. The experiments and sample collection were performed at ZT20; (**B**,**C**) the contents of plasma total cholesterol and plasma triglyceride (TG) in the *db/db* mice. The mice were treated the same way as (**A**) (*n* = 3−4 per group); (**D**) the levels of liver TG and TC in the *db/db* mice (*n* = 3−4 per group); (**E**) mRNA levels of *Bmal1*, *Per2*, *Srebp1c*, and *Fasn* analyzed by real−time qPCR in the animal livers (*n* of animal = 3−4 per group); (**F**) Western blots of BMAL1, DBP, and FASN proteins in the animal livers (n of animals = 3 per group). * *p* < 0.05; ** *p* < 0.01.

The decrease in BMAL1 expression and the impacts of APC on lipid and glucose metabolism could be correlated. To identify such a correlation, we injected an adenovirus expressing Bmal1 to reverse the decrease of APC−inhibitory BMAL1 expression in the livers of APC− or vehicle−treated *db/db* mice (Figure 7A) and examined its effect on lipid metabolism. Interestingly, APC reduced plasma/hepatic TC and TG levels in *db/db* mice, but the reduction in plasma/hepatic lipid levels was counteracted in BMAL1−overexpressed mice (Figure 7B–D). Similar to the in vitro results, hepatic BMAL1 overexpression in vivo mitigated the APC−induced inhibition of mRNA and protein levels of relevant genes in hepatic lipid synthesis (Figure 7E,F). Overall, these results indicated that APC inhibits hepatic lipid synthesis in the presence of BMAL1. Subsequently, we investigated the effects of APC on glucose metabolism in *db/db* mice overexpressing BMAL1 in the liver. An insulin tolerance test (ITT) revealed that hepatic BMAL1 overexpression did not affect insulin sensitivity in APC−treated mice (Figure S2A), and a pyruvate tolerance test (PTT) showed that overexpression of BMAL1 did not affect the inhibitory effect of APC on gluconeogenesis in *db/db* mice (Figure S2B). The fasting blood glucose level remained unaffected in APC−treated mice after overexpression of BMAL1, which was consistent with the PTT results (Figure S2C). In general, these results indicated that APC may inhibit hepatic lipid synthesis in the presence of BMAL1 and modulate hepatic lipid metabolism in a BMAL1−dependent manner. Moreover, APC could also inhibit the transcriptional activity of BMAL1 by regulating the CREB/CRTC2−BMAL1 signaling pathway.

## 4. Discussion

APC has been verified to relieve metabolic syndrome in obese mice by blocking the CREB/CRTC2 complex [8]. In the present study, APC treatment showed a significant impact on glucose and lipid homeostasis, with ZT17 APC administration having increased impact on glucose and lipid metabolism compared to ZT5 APC administration (Figure 1). Our genetic and pharmacological data revealed that APC inhibits hepatic lipid metabolism by suppressing the transcriptional activity of BMAL1. BMAL1 overexpression in the liver could antagonize the inhibitory effect of APC on hepatic lipid synthesis in *db/db* mice (Figure 6). Our study highlighted that APC exerts its effects in a BMAL1−dependent manner. In fasting conditions, CRTC2 was found to be recruited to the *Bmal1* promoter to induce *Bmal1* expression [41]. In this report, CRTC2 overexpression in primary mouse hepatocytes confirmed that APC inhibits BMAL1 transcription by blocking the formation of the CREB/CRTC2 (Figure 2). In conclusion, our results demonstrated that APC could alleviate metabolic syndrome in obese mice in a circadian rhythmic manner.

To reveal the effects of APC on the expression of BMAL1, we detected the endogenous protein levels of BMAL1 in primary hepatocytes, as well as the activity of Bmal1−luc in 293T cells overexpressing Bmal1−luc. Our data showed that the effect of APC on endogenous BMAL1 expression seems to be smaller than its effect on Bmal1−Luc. We believe that the effect of APC on endogenous expression is a physiological effect, whereas the effect on overexpression is not (Figure 2B,E).

Numerous studies have found that the 24 h rhythmic expression of a few genes could lead to distinct drug efficacies [10,42]. Similarly, our results suggest that APC significantly alters glucose and lipid metabolism by inhibiting BMAL1 expression at ZT20, the time point when its transcriptional activity is elevated. By contrast, at ZT8, when the BMAL1 transcriptional activity was limited, the efficacy of APC was not significant. Although we verified the essential role of BMAL1 in the circadian rhythmic effect of APC on hepatic glucose and lipid metabolism, more studies are needed to reveal the importance of APC absorption and metabolism.

As a key member of the core molecular circadian clock, BMAL1 regulates both the circadian clock and energy homeostasis [26,28,43]. Several studies have demonstrated the critical role of BMAL1 in the regulation of glucose and lipid metabolism. For example, mice with *Bmal1* deleted in the intestine are less vulnerable to obesity even on a high−fat diet (HFD) but have a normal phenotype on a chow diet [44]. Furthermore, during refeeding, liver−specific overexpression of BMAL1 in mice could promote de novo lipogenesis by activating the insulin−mTORC2−AKT signaling [45]. As is described above, APC was observed to inhibit hepatic de novo lipogenesis in the liver by inhibiting the transcriptional activity of BMAL1, and such inhibition was antagonized by BMAL1 overexpression, suggesting that BMAL1 has an essential role in de novo hepatic lipogenesis.

Genetic and pharmacological studies have revealed that BMAL1 plays a complex role in hepatic lipid synthesis and glucose metabolism. Our findings show that APC decreases gluconeogenesis and increases insulin sensitivity. Unlike the situation during hepatic lipid synthesis, overexpression of BMAL1 did not affect the APC−mediated inhibition of glucose metabolism. Our results showed that the effects of APC on glucose metabolism may be partially independent of BMAL1 (Figure S2).

APC was found to prevent metabolic syndrome in obese mice because of its ability to disrupt CREB/CRTC2 interactions [8]. This study demonstrated that APC had minimal effect on WT mice compared to obese ones. No significant changes in fasting blood glucose and plasma TG levels were observed in APC−treated WT mice under a recipe of once daily for two weeks, suggesting the safety of APC.

## 5. Conclusions

In conclusion, our study revealed that APC could alleviate metabolic syndrome in obese mice, which is influenced by circadian rhythm, and the efficacy of APC could be enhanced by night−time administration. Furthermore, APC may improve hepatic glucose and lipid metabolism by regulating the CREB/CRTC2−BMAL1 signaling pathway. These findings may prove useful in understanding the circadian rhythmic effects of APC on metabolic syndrome and maximizing the benefits of APC in promoting metabolic health.

## Data Availability

All relevant data are within the manuscript and its Supporting Information Files.

## Supplementary Materials

The following supporting information can be downloaded at: https://www.mdpi.com/article/10.3390/nu15071644/s1, Figure S1: APC inhibits hepatic BMAL1 and PER1 expression; Figure S2: Overexpression BMAL1 in the livers of *db/db* mice did not affect APC−mediated glucose homeostasis. Table S1: Primers of Real−Time PCR; Table S2: Primary antibodies for western blotting.
