# Supplementary material for: Artepillin C Time−Dependently Alleviates Metabolic Syndrome in Obese Mice by Regulating CREB/CRTC2−BMAL1 Signaling
Source: Nutrients. 2023 Mar 28;15(7):1644. doi: 10.3390/nu15071644 (PMC10096790; doi:10.3390/nu15071644)
Supplement: Supplementary file 1 [file nutrients-15-01644-s001.zip › nutrients-2229893-supplementary.pdf]

Figure S1.

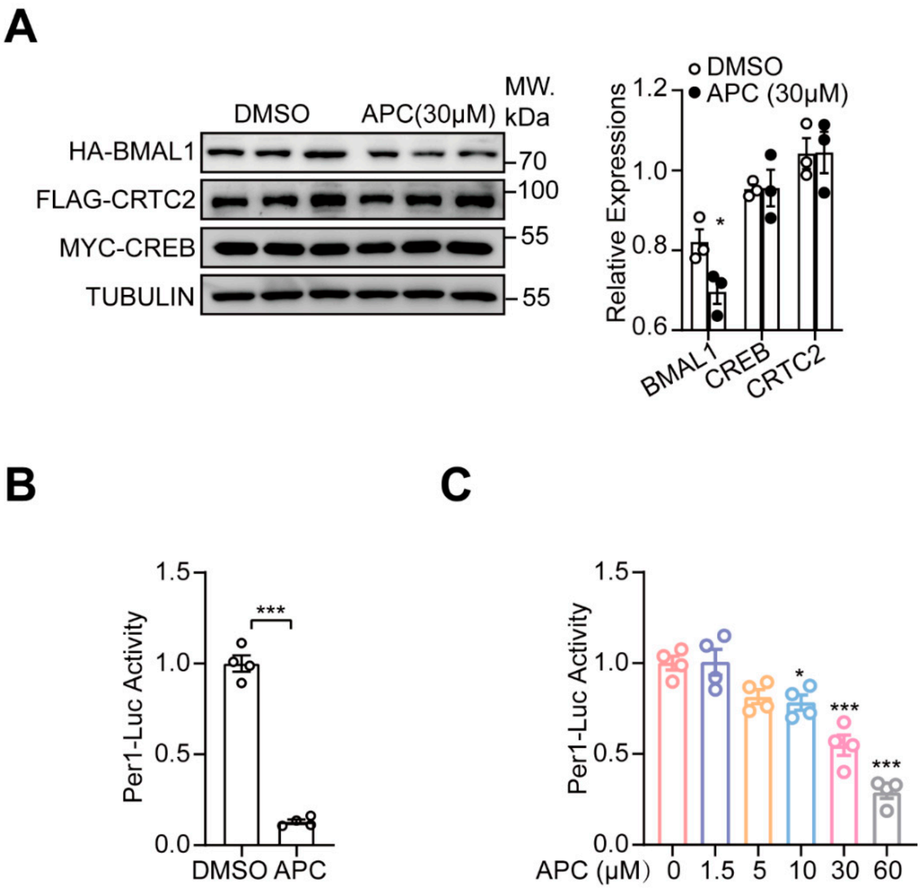

Figure S1. APC inhibits hepatic BMAL1 and PER1 expression. (A) Immunoblotting of overexpression protein levels of HA-BMAL1, FLAG-CRTC2, MYC-CREB, and TUBULIN in HEK 293T cell treated with DMSO or APC (30 μM) for 1 h prior to 7 h stimulation with forskolin (100 nM, *n* = 3 per treatment). (B) Luciferase Per1-Luc activity. HEK293T cell were infected by Per1-Luc and RSV-β-Gal for 24 h, then incubated with APC (30 μM) for 1h prior to forskolin 7 h before luciferase assay (*n* = 4 per treatment). (**C**) Primary hepatocytes were infected by AD-Per1-Luc and AD-RSV-β-Gal for 24 h, then incubated with APC (0, 1.5, 5, 10, 30, 60 μM) for 1 h prior to 7 h stimulation with glucagon (100 nM, *n* = 4 per treatment) Different colors represent different group treated with different concentrations of APC. * $p < 0.05$; ** $p < 0.01$; *** $p < 0.001$

Figure S2.

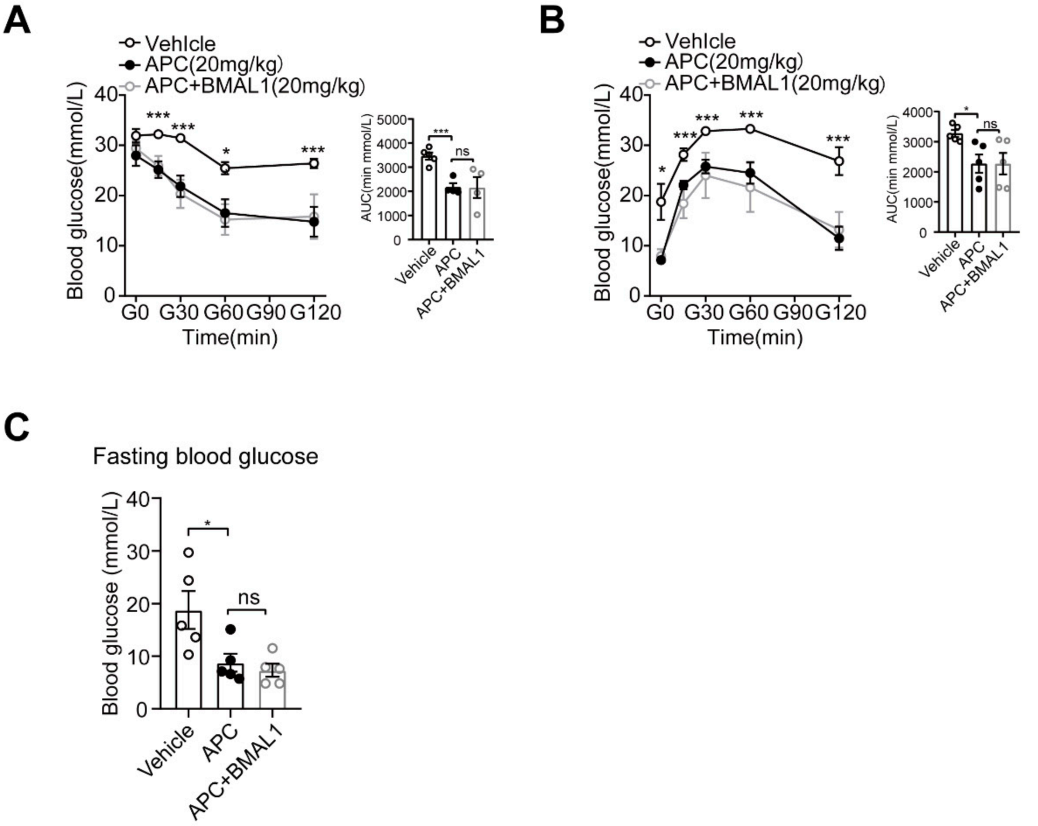

Figure S2. Overexpression BMAL1 in the livers of *db/db* mice did not affect APC-mediated glucose homeostasis. (A) Insulin tolerance test (ITT) and (B) pyruvate tolerance test (PTT) of *db/db* mice administrated APC or Vehicle for two weeks at ZT 20. The *db/db* mice was injected with adenovirus overexpressing BMAL1 or GFP. The results of area under curve (AUC) are shown at the bottom of each test curve (n = 4-5 per group). (C) The fasting blood glucose levels. The *db/db* mice were treated by APC (20 mg/kg) for 2 weeks (n = 5 per group). * $p < 0.05$; ** $p < 0.01$; *** $p < 0.001$

Table S1. Primers of Real-Time PCR

Gene	qPCR primers
Bmal1	5’- TGCAGAACACCAAGGAAGGA -3’
	5’-ATTTTGTCCCGACGCCTCTT-3’
Dbp	5’-CTGGCCCGAGTCTTTTTGC-3’
	5’-CCAGGTCCACGTATTCCACG-3’
Per1	5’- AGCAGGACAACCCATCTACCA-3’
	5’- CGAAGTTTGAGCTCCCGAAGT-3’
G6pc	5’- TCTGTCCCGGATCTACCTTG -3’
	5’- GTAGAATCCAAGCGCGAAAC -3’
Pepck	5’- GTGCTGGAGTGGATGTTCGG -3’
	5’- CTGGCTGATTCTCTGTTTCAGG -3’
Srebp1c	5’- GCGGAGCCATGGATTGCAC-3’
	5’- CTCTTCCTTGATACCAGGCCC-3’
Srebp2	5’- GCGTTCTGGAGACCATGGA-3’
	5’- ACAAAGTTGCTCTGAAAACAAATCA-3’
Fasn	5’- GCTGCGGAAACTTCAGGAAAT-3’
	5’- AGAGACGTGTCACTCCTGGACTT-3’
Scd1	5’- CCGGAGACCCCTTAGATCGA -3’
	5’- TAGCCTGTAAAAGATTTCTGCAAACC-3’
Acc	5’- TGACAGACTGATCGCAGAGAAAG-3’
	5’- TGGAGAGCCCCACACACA-3’
L32	5’- TCTGGTGAAGCCCAAGATCG -3’
	5’- CTCTGGGTTTCCGCCAGTT -3’
Crtc2	5’- CACCAGAACTTGACCCACTGT -3’
	5’- CACAGGGGTCACTCAGCATAG -3’

Table S2. Primary antibodies for western blotting

Antibodies name	Company	Dilution
BMAL1	Abcam, ab3350	1:1000
PER1	Abclonal, A8449	1:1000
DBP	Abcam, ab22824	1:1000
HA	CST, 3724	1:2000
Myc	CST, 18583	1:2000
M2 Flag-HRP	Sigma, A8592	1:4000
SREBP1(2A4)	Santa cruze, sc-13551	1:2000
ACC	Beyotime, AF1867	1:2000
SCD	Abcam, ab19862	1:1000
FASN	Beyotime, AF5168	1:1000
ACTIN	Abmart, T40104	1:8000
TUBULIN	Abmart, T40103	1:8000
GAPDH	AGOMA, AGM90111	1:8000
PEPCK(H-300)	Santa cruze, sc-32879	1:1000
G6pase	Proteintech,22169-1-AP	1:1000
CRTC2	Millipore, ST1099	1:8000
CREB	CST, 9197	1:1000
Phospho-CREB(S133)	CST, 9198	1:1000
